# Burnout, Drop Out, Suicide: Physician Loss in Emergency Medicine, Part I

**DOI:** 10.5811/westjem.2019.4.40970

**Published:** 2019-04-23

**Authors:** Christine R. Stehman, Zachary Testo, Rachel S. Gershaw, Adam R. Kellogg

**Affiliations:** * Indiana University School of Medicine, Department of Emergency Medicine, Indianapolis, Indiana; † University of Massachusetts Medical School-Baystate Health, Department of Emergency Medicine, Springfield, Massachusetts

## Abstract

Each year more than 400 physicians take their lives, likely related to increasing depression and burnout. Burnout—a psychological syndrome featuring emotional exhaustion, depersonalization, and a reduced sense of personal accomplishment—is a disturbingly and increasingly prevalent phenomenon in healthcare, and emergency medicine (EM) in particular. As self-care based solutions have proven unsuccessful, more system-based causes, beyond the control of the individual physicians, have been identified. Such system-based causes include limitations of the electronic health record, long work hours and substantial educational debt, all in a culture of “no mistakes allowed.” Blame and isolation in the face of medical errors and poor outcomes may lead to physician emotional injury, the so-called “second victim” syndrome, which is both a contributor to and consequence of burnout. In addition, emergency physicians (EP) are also particularly affected by the intensity of clinical practice, the higher risk of litigation, and the chronic fatigue of circadian rhythm disruption. Burnout has widespread consequences, including poor quality of care, increased medical errors, patient and provider dissatisfaction, and attrition from medical practice, exacerbating the shortage and maldistribution of EPs. Burned-out physicians are unlikely to seek professional treatment and may attempt to deal with substance abuse, depression and suicidal thoughts alone. This paper reviews the scope of burnout, contributors, and consequences both for medicine in general and for EM in particular.

## INTRODUCTION

“Burnout” evokes images of harried, sleep-deprived, hungry physicians, overwhelmed with “paperwork,” administrative complaints of missed metrics, and pending tasks for family and patients. For the physician suffering from burnout, recovery can seem daunting or even impossible. For healthcare, burnout has been branded an epidemic, with societal and human economic and personal costs.^1^ This article, the first of two parts, synthesizes information on burnout—the scope of the problem, its causes and consequences—from the perspective of the emergency physician (EP). Part II will focus on wellness and seek to make recovery less daunting.

### Burnout: Definition and Measurement

Burnout is a complex condition with a history in many disciplines. Based on his research, Freudenberger used “burnout” as shorthand for a psychological syndrome with three dimensions: emotional exhaustion, depersonalization, and reduced personal accomplishment.^2^ Maslach subsequently summarized the dimensions of burnout as “exhaustion,” “cynicism,” and “inefficacy,” providing more identifiable definitions of each dimension that align well with her measurement tool.^3^ Those who score high in “exhaustion” feel over-extended, their emotional and physical resources depleted.^3^ High scorers in “cynicism” (depersonalization) appear more callous or detached than would be expected for normal “coping.”^3^ Those lacking confidence or feeling they have achieved little work success score high in the “inefficacy” (reduced personal accomplishment) dimension.^3^ Overall, sufferers from burnout are frequently exhausted, diminished in their ability to care, and feel as though their work makes little difference.

Maslach used these definitions to create the most frequently used assessment tool for identifying burnout, the Maslach Burnout Inventory (MBI). This tool contains 22 questions addressing the three dimensions and provides scores in each. The higher the score, the higher the burnout in that dimension.^4^ Rather than a dichotomous cutoff score of burnout as a diagnosis, the MBI describes a spectrum with higher scores equating to more severe symptoms and consequences.^5^ While the MBI has been modified and abbreviated for specific populations and ease of use, it remains proprietary. The next most common tool used in healthcare burnout research, the Oldenburg Burnout Inventory, focuses on emotional exhaustion and depersonalization/disengagement, while leaving out personal accomplishment.^6^ A list of burnout assessment tools appears in Appendix 1; however, readers may consider simply asking physicians if they are burned out: In one study, self-reported burnout accurately predicted meeting MBI burnout criteria 72% of the time.^7^

## METHODS

### Keywords

We chose “burnout” and its main components (“emotional exhaustion,” “depersonalization,” “cynicism,” “job dissatisfaction”) as the endpoint keywords. Because healthcare burnout researchers leave out the “lack of personal accomplishment” dimension, we did the same here.^8^ “Depression” and “suicide,” the ultimate consequences of burnout, were also included as endpoints. These keywords were paired with population keywords: “physicians,” “residents,” “medical students,” and “emergency medicine” (EM) to find relevant articles in the medical literature.

### Search

We searched all combinations of pairings of each “endpoint” keyword with a “population” keyword from 1974 to the present in both Ovid Medline and PubMed. To ensure more esoteric sources were included we conducted searches for “endpoint” keywords on various EM/critical care blogs and lay press Web sites.^9^

### Article Inclusion Criteria

We categorized all search results into primary research studies, commentary/opinion pieces, and review articles. Primary research studies, inclusive of their relevant references, provided the database of supporting information for the composition of the review. Additionally, we attempted to identify the primary literature for all Internet-based resources.

## RESULTS

### Scope of Burnout in Physicians

Freudenberger and Maslach initially identified and studied burnout in non-medical fields; however, as early as 1981, research began to focus on burnout in physicians and medicine.^10^ In 2012 a landmark study identifying burnout as high scores in either the MBI’s depersonalization or emotional exhaustion dimension found that 37.9% of physicians met criteria for burnout compared to 27.8% of the general United States (U.S.) workforce.^8^ Since 2013, Medscape has published the results of an annual survey of physicians. Per this report, the percentage of physicians experiencing burnout has steadily risen.^11^ Most recently, 44% of respondent physicians indicated feeling burned out, a percentage that correlates with the most recent survey by Shanafelt et al. (43.9% respondents had at least one symptom of burnout).^11^,^12^

Burnout has been studied at all levels of medical training and starts early: one study identified 52.8% of students (an equal mix of all four years) from seven medical schools meeting criteria.^13^ Burnout continues during residency, though it has been less frequently explored. In 2002 Shanafelt et al. found that 76% (n = 87/115) of one internal medicine program’s residents met criteria for burnout.^14^ In a 2018 study, researchers surveying 3588 second-year resident physicians across multiple specialties found that 45.2% experienced at least one symptom of burnout at least weekly.^15^ A recent systematic review and meta-analysis aggregated 26 studies including 4664 residents of multiple specialties and found a burnout prevalence of 35.7%, consistent with previous work.^16^ This early-career burnout seems to predict later-career burnout, as suggested by a small study of internal medicine residents (N = 81) over 10 years.^17^ They found high univariate correlations between emotional distress in residency and later emotional exhaustion (correlation coefficient=0.30, P = 0.007) and depersonalization (correlation coefficient=0.25, P = 0.029).^17^ For an expanded list of different burnout and wellness surveys and scales, please see Table 1 in the Appendix.

### Causes of Burnout

Historically, medicine saw burnout as a sign of personal weakness or of being ill-suited to the profession.^18^ Without consideration of organizational and societal influences on burnout development, authors suggested that “self-rescue” would occur if one simply recognized his or her condition and engaged in improved communication and management-skills training or routine exercise.^19^–^21^ Even leading researchers espoused these beliefs: Shanafelt et al. stated that physician burnout was related to stressful work, doing too much and putting others’ needs before their own.^22^ However, the results of Shanafelt’s landmark 2012 study on the prevalence of burnout appeared to have changed his views, and he called on others to take a different perspective:

*“The fact that almost 1 in 2 U.S. Physicians has symptoms of burnout implies that the origins of this problem are rooted in the environment and care delivery system rather than in the personal characteristics of a few susceptible individuals.”*^8^

Although individual characteristics do contribute to burnout susceptibility, and physicians cope with burnout using exercise and meditation, the problem has not improved.^11^,^23^ Individual physicians seem to recognize the importance of outside forces on their experience of burnout, even if society and organizations have not fully embraced this. The responses to the yearly Medscape survey now lists only organizational and environmental causes for burnout, such as bureaucratic tasks, long work hours, electronic health records (EHR), lack of respect, lack of control/autonomy, and profits over patients.^11^ The following discussion will focus on three contributing factors: EHRs, financial concerns, and the “second victim” syndrome (SVS).

#### Electronic Health Records

While charting was once used to communicate relevant clinical information between members of the healthcare team, the EHR has shifted medicine’s focus to billing, coding, and protection from litigation. EHRs are independently associated with higher rates of burnout among users.^24^ Clinical time spent more on the computer than with patients impairs patient contact (ie, “the best part of being a doctor”). Less one-on-one time with patients leads to a decrease in humanism and conflicts with physicians’ inherent altruism. This in turn increases the risk of burnout and substantiates the views of the Medscape respondents: profits over patients.^25^, ^26^

EHRs impact physician workflow as time-consuming distractions that create new problems, such as downtimes and electronic-prescription system failures. Downtimes are typically scheduled at “slow times” for the hospital in the middle of the night, when EPs and emergency departments (ED) are often busiest and staffing scarce. The EHR’s billing-centric design slows chart-completion, and online availability can lead to uncompensated charting at home.^26^,^27^ While physicians generally agree that EHRs have improved access to medical records and provide some benefits, they decrease patient interaction, worsen work-life balance, and decrease job satisfaction, resulting in overall net harm to physicians.^27^

#### Financial Concerns

While Medscape respondents mention “lack of compensation/reimbursement,” their concerns may be tied to medical school debt.^11^ The cost of medical education continues to rise; physicians who graduated in 2016 carry an average debt over $190,000, which correlates with burnout.^28^,^29^ Additionally, physicians feel under-prepared to navigate their finances while transitioning to attending-level income.^30^ This lack of preparation may lead to living above their means, worsening their debt despite high income, resulting in increased stress and burnout.^31^

#### Second Victim Syndrome

Another likely contributor to *and* consequence of burnout is the SVS phenomenon.^34^–^36^ SVS embodies the psychological trauma healthcare workers suffer from involvement in an “adverse event.” Typically related to committing a medical error resulting in a poor patient outcome, SVS may also involve any adverse patient outcome, expected or unexpected, with the physician becoming the “second victim.”^37^ One study found that 30% of physicians (all specialties) experienced emotional issues related to a “bad outcome,” while another found up to 60% of surgical residents experienced SVS.^38^, ^39^

Society sets a zero-mistake standard for physicians.^32^ This high standard may isolate those who make mistakes leaving them without healthy ways to cope, resulting in dysfunctional approaches to recovery.^32^,^35^,^36^ Poor responses (isolation, anger, sadness, substance abuse, and callousness toward patients and colleagues) place the physician more at risk for burnout.^35^,^36^ When suffering from SVS, the perception of not being supported or even of being victimized by one’s own hospital or organization can exacerbate the syndrome.^32^,^40^ This sense of victimization comes despite research suggesting that medical errors leading to poor patient outcomes stem from system failures and not just the individual who committed the error.^41^,^42^ This is a continuous chain of events; if a physician is burned out, he or she is more likely to commit an error during patient care, which puts them at risk for SVS and litigation stress and likely exacerbates their burnout.^32^,^36^,^43^–^45^ This cycle and its associated emotional toll lead to negative consequences, which may include depression and departing medicine by either attrition or suicide.^35^

### Consequences of Burnout

Additional consequences of burnout include poor clinical care, increased mistakes, patient dissatisfaction, dysfunctional interactions between colleagues, the contagion of burnout, substance abuse/self-medication, depression, and suicide.

#### Clinical Care

Health systems now recognize the negative impact of burnout on healthcare quality, patient safety, and financial performance.^46^ A study of U.S. surgeons found both an increased rate of medical errors and greater medicolegal risk for physicians experiencing burnout.^47^ A recent meta-analysis found a statistically significant negative relationship between physician burnout and patient safety (r = −0.23), as well as burnout and quality of care (r = −0.26).^48^ As clinical care suffers, so does patient satisfaction, which in turn may further decrease health outcomes.^49^,^50^ Burnout may also affect a physician’s colleagues by being contagious: burned-out physicians negatively interact with co-workers and perform more poorly at their jobs, creating a negative work environment and putting others at risk for burnout.^3^,^51^,^52^

#### Leaving Jobs/Medicine

Physicians suffering burnout are significantly more likely to leave healthcare.^53^,^54^ Physicians first reduce work hours or change jobs or specialties, negatively affecting the health system. The estimated cost to replace a physician is $160,000–$1,000,000, depending on specialty and experience. This estimate does not include intangibles such as team disruption.^11^,^46^,^55^–^57^ If this job change does not help, physicians may seek administrative positions or leave medicine entirely.^58^

#### Depression and Self-medication

Burnout occurs on a continuum with depression. The 2012 study by Shanafelt et al. found that 37.8% of respondents screened positive for depression on a standardized and validated two-question screening tool.^8^ The most recent Medscape survey indicated that 15% are not only burned out, but also are either “colloquially” or clinically depressed.^11^ Multiple barriers separate physicians from depression assistance. Such barriers include feeling that they do not require professional intervention and, perhaps more importantly, fearing the loss of medical licensure and hospital credentialing.^11^,^59^ A 2014 survey found that nearly 40% of physicians would be reluctant to seek care for mental health due to licensure concerns.^60^

While many physicians deal with burnout and depression in isolation, some have developed harmful coping strategies such as alcohol and drug use.^11^ In general, older research suggests that approximately 10–12% of physicians will develop at least one substance abuse disorder, similar to the general population rate.^61^ More recent data suggest physicians primarily abuse alcohol, with 12.9% of male physicians and 21.9% of female physicians affected, numbers higher than the general population. (Overall 6.2% of the U.S. population 18 years or older has an alcohol use disorder, 8.4% of men and 4.2% of women.)^62^,^63^

#### Suicide

Society is shocked when a physician commits suicide. It is estimated that 400 physicians in the U.S. die by suicide each year.^64^ Compared to the general population, male and female physicians are at greater relative risk (RR) of suicide (RR = 3.4 and RR = 5.7, respectively).^65^,^66^ Shanafelt, et al. reported that 6.4% of respondents had considered suicide in the previous year.^8^ In the most recent Medscape report, 14% of respondents had considered suicide and 1% of respondents had attempted suicide, results similar to a study of female physicians (1.5% attempted suicide).^11^, ^67^ Physicians in training are not immune to these risks. Approximately 10% of medical students report suicidal ideation, and suicide is the second leading cause of death among resident trainees in the U.S. (4.1 per 100,000, or approximately five residents per year).^68^–^70^

While these rates of physician depression and suicidal ideation do not significantly differ from those of the general working population (37.8% and 6.4%, respectively), there are reasons to believe that physician depression is both under-reported and under-treated.^8^ Physicians are less likely to seek treatment since depression remains stigmatized in medical culture.^41^,^71^ Depressed physicians may feel like failures, isolated and cut off from their colleagues whom they believe are coping better. Feelings of isolation, loss of belonging, and failure, combined with the perception of being a burden on partners, family, friends and society, drive some to see suicide as an answer.^72^

Given that physicians do not seek help and approximately one in seven has considered suicide, someone reading this may be suffering from depression and contemplating suicide. If that is you, please reach out to a friend, a helpline (call 1-800-273-8255 or text HOME to 741741), a therapist, or to an employee assistance program. Anyone with concerns that a colleague is suffering should reach out, ask, listen, and assist him or her in finding help. For a comprehensive list of suicide prevention and self help resources, please see Table 2 in the Appendix.

## DISCUSSION

In medicine, EM is unique in its hours, patient population and stressors. This uniqueness translates into more EP burnout. A four-year survey published in 1996 found that 60% of EP respondents “registered in the moderate to high burnout ranges” on the MBI.^7^ In the 2012 landmark burnout study, EM was the most burned-out specialty (~65%), over 10% more “burned out” than the next closest specialty (general internal medicine), and close to 20% more than the mean rate for all physicians responding.^8^ While burnout in EM has continued, the most recent Medscape report indicates that EM is the fifth most burned-out specialty behind urology, neurology, physical medicine and rehabilitation, and internal medicine.^11^ Like other specialties, burnout in EM starts early, with studies showing between 65–74% of residents (all levels) meet criteria for burnout.^73^,^74^

### Causes of Burnout in Emergency Medicine

The unique stressors in EM may easily lead EP burnout to be attributed to personal characteristics such as poor coping skills or lack of exercise, rest, and hobbies, a view that continues to this day. However, organizational and environmental causes of burnout certainly apply to EPs. One notable exception is the usual connection between burnout and increased work hours. For non-EPs, burnout appears to directly correlate with increasing work hours.^11^ On the contrary, while EPs are the least likely specialists to work excessive hours (>40 hours/week), the necessity of working nights and on weekends and holidays may contribute to burnout.^11^ Furthermore, the lack of support staff and medical infrastructure during these “off” hours, coupled with high intensity work (heavy workload, multiple sick patients, frequent task-switching, patient and colleague rudeness, and constant uncertainty) may have a similar effect on EP emotional health as the longer hours of other specialists.^75^–^79^

With fewer weekly hours than other specialties, EPs have the ability to “pick up” extra shifts, increasing their work hours and the associated stress. Many EPs work extra shifts to pay off debt, another stressor and contributor to burnout.^80^ In 2016 the median debt of EM residents in one study was $212,000.^81^ This debt caused stress and changed plans: getting out of debt reportedly took priority over pursuing further educational opportunities, vacations, and spending time with family, all things that might counter burnout.^80^,^81^ The ability to “pick up” extra shifts to pay down debt and the perception that they are working less than other physicians are examples of particular attributes of EM that increase susceptibility to burnout.^82^ Three other causes of burnout in EM deserve mention: clinical pressures/expectations, litigation stress, and fatigue/sleep loss.

#### Clinical Pressures and Expectations

Society perceives EM as a world of excitement, drama, and miraculous saves.^83^ While not wholly inaccurate, television dramas do not show the persistent demand for immediate and error-free care despite limited resources.^84^ This mismatch between demands and resources, coupled with constant diagnostic uncertainty, significantly stresses EPs and promotes burnout and emotional exhaustion.^79^,^84^–^86^

Both EDs and EPs are limited resources: EDs are closing while visits are increasing, and there is a national shortage of EPs, particularly in less geographically desirable areas.^87^,^88^ Despite a consistent increase in EM first-year residency training positions (1786 in 2014 to 2278 in 2018, 27.5% increase), only 61% of U.S. emergency care providers are EPs, with the rest a combination of advanced practice providers (APPs) (24.5%) and non-EPs (14.3%).^89^, ^90^ This shortfall particularly affects rural areas where only 44.8% of rural emergency care providers are EPs.^90^ Despite this shortfall, EPs provide care for 85.3% of ED patients, meaning they are working more clinical hours while being responsible for care being provided by APPs.^88^, ^91^

Compensation is often based on productivity, patient satisfaction, and “quality” measures.^92^ With more patients and less time to see them, EPs who are judged on patient satisfaction may choose to acquiesce to requested, but not medically indicated, care. This occurs despite patient satisfaction correlating poorly with quality of care.^93^–^97^ Similarly, the guidelines and care metrics nominally designed to improve patient care (eg, door-to-doc/needle/antibiotics time) are rigorously enforced despite lack of evidence of patient benefit.^98^–^99^ Such metrics and guidelines, particularly prominent in EM as the initial provider of care, deprive physicians of autonomy and the ability to practice the art of medicine, leading to job dissatisfaction and burnout.^82^, ^100^

#### Litigation Stress

Being the first care provider for so many sick patients means inevitably dealing with a malpractice claim, another cause of burnout.^101^ Annually, EPs face malpractice claims at a slightly higher rate than the average physician (8.7% vs 7.2%).^102^ Each litigation episode can last years, and physicians are counseled not to discuss such cases with anyone, adding to the isolation and lack of peer support.^103^,^104^ Annually, up to 73% of EPs admit to practicing “defensive medicine,” ordering extra tests to avoid missing anything, and cite fear of litigation as the reason.^105^ This practice leads to physician cynicism and disengagement (precursors to burnout), and increases healthcare spending (by an estimated $750 billion in 2010).^106^

#### Sleep loss and fatigue

One reason EPs likely face higher litigation rates is that they simply encounter more sick patients than other physicians, as their work environment is available at all times. To fulfill the 24-hour need for high quality emergency care, EM is built around shift work. The resulting disruption of circadian rhythms leads to sleep loss and its associated detrimental effects on health: increased cardiovascular disease, metabolic syndrome, sleep disorders, and possibly even increased mortality.^107^,^108^ The effects of shift work are felt early (84% of five cohorts of EM residents felt a need for intervention for their sleep deprivation and self-perceived exhaustion) and become more pronounced with age.^109^,^110^ Sleep deprivation is associated with worse patient care, decreased job satisfaction, and less personal well-being, all of which contribute to burnout.^111^

### Consequences of Burnout in Emergency Medicine

While the consequences of burnout for EPs are similar to those for physicians in general, certain areas deserve specific mention: clinical care, depression, substance abuse, SVS, and suicide.

#### Clinical Care

Like other physicians, burned-out EPs self-report delivering suboptimal clinical care and more often perceive they have erred medically.^73^ Such EPs also have lower patient satisfaction scores and perform worse during high-fidelity simulations compared with their peers who are not burned out.^111^, ^112^

#### Physician Drop Out

Although attrition from EM has historically been low (1.7% per year, in a 2010 study), attrition rates do not account for those feeling “trapped” in their current jobs due to debt.^81^,^113^ This may be one reasons why EPs are the second least happy at work behind physical medicine and rehabilitation.^11^ EPs may forego further training or changing jobs due to debt, creating a feeling of hopelessness that further contributes to stress and burnout.^81^ Ironically, further training in a subspecialty of EM could serve to reduce burnout by adding variation to an EP’s work schedule and duty.^114^

#### Depression

Researchers have found rates of depression in EPs (12.1% – 19.3%) consistent with the Medscape survey of depression rates in all respondents (11–15%). 11,115–116

#### Self-medication

Both EPs and EM residents experience higher rates of substance abuse than other specialties, with studies estimating that 4.9–12.5% of EM residents drink daily.^116^,^117^ Other research suggests that 7–18% of the physicians treated for substance abuse are EPs, despite only 4.7% of all physicians being EPs.^118^–^120^

#### Second Victim Syndrome

While no specialty-specific numbers exist, EPs seem especially susceptible to SVS. EPs rarely have time to debrief or grieve after an adverse patient outcome, because there is always the next patient.^37^ Most EPs have a story about a patient dying despite their best efforts and then having to see a lower acuity patient unhappy because of an extended wait. This lack of processing time for patient deaths or medical errors may make EPs more susceptible to SVS and, by extension, burnout. Conversely, burned-out physicians are more likely to commit a medical error and have poorer job-coping skills. SVS is complex and intimately tied to depression and burnout, with all three contributing to and resulting from the others.^37^ However, they are related: SVS, burnout, and depression may all result in an EP leaving the specialty in the most final way – suicide.

#### Suicide

While no specialty-specific data exists and the Medscape data may contain biased responses, extrapolation from that data suggests that, in the last year, as many as 6,000 EPs have contemplated and up to 400 have attempted suicide.^11^,^121^ The following factors may explain why these numbers are so high: (a) EM seems to have a higher rate of gender-based harassment of women (45.3% vs 20.3%) than the medicine average;^122^ (b) female physicians have a much higher rate of suicide than their general population counterparts (130% higher);^123^ (c) there is an association between workplace harassment, depression and suicide;^122^ and (d) physicians tend to “succeed” in their suicide attempts more often than the general population.^72^

## CONCLUSION

**Figure 1:**
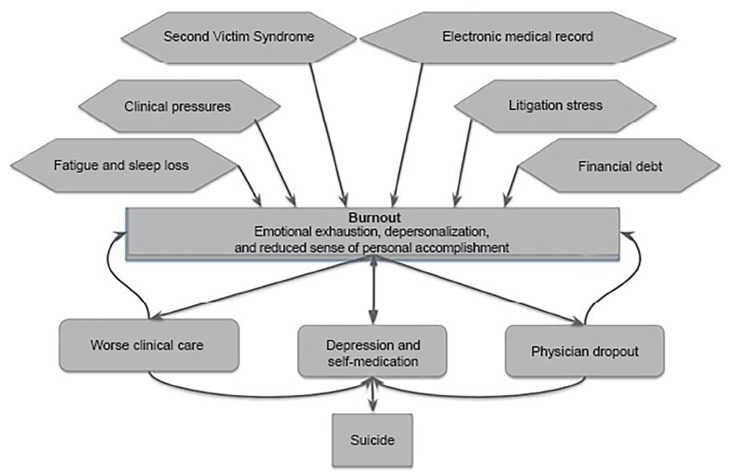
The causes and consequences of physician burnout.

While suicide is its ultimate tragic outcome, burnout is a complex condition resulting in many consequences. Since EPs are particularly vulnerable to burnout due to the system, culture and society in which they practice, we need to understand the complicated interaction between the signs, symptoms, causes, and consequences of burnout (Figure 1). This understanding can help create a path to recovery, both individually and as a specialty. As practitioners of a specialty who experience burnout at such high levels, EPs should take the lead in this recovery. Resources to aid in recovery will be found in Part II of this series, which discusses mitigating burnout and its consequences through *wellness*, “the anti-burnout.”

## Supplementary Information
