# Supplementary material for: Burnout, Drop Out, Suicide: Physician Loss in Emergency Medicine, Part I
Source: West J Emerg Med. 2019 Apr 23;20(3):485–94. doi: 10.5811/westjem.2019.4.40970 (PMC6526882; doi:10.5811/westjem.2019.4.40970)
Supplement: Supplementary file 1 [file wjem-20-485-s001.docx]

Table 1. Burnout, Satisfaction and Competency Scales: Measures of physician burnout, well-being, job satisfaction and related competencies available to physicians and departments.

| Name | Target Audience | Description | Resources |
| --- | --- | --- | --- |
| Maslach Burnout Inventory | Human services professionals | Anonymous individual survey measuring burnout across three dimensions: depersonalization, emotional exhaustion and reduced personal accomplishment. | [www.mindgarden.com/117-maslach-burnout-inventory](https://www.mindgarden.com/117-maslach-burnout-inventory) |
| Emotional and Social Competency Inventory | Professionals geared towards high performance | Inventory assessing 12 competencies of social and emotional intelligence over four areas of ability: social and self-awareness and relationship and self-management. | [www.eiconsortium.org/measures/eci_360.html](http://www.eiconsortium.org/measures/eci_360.html) |
| Emergency Physician Job  Satisfaction Measure | Emergency Physicians | Instrument to evaluate the job satisfaction of Emergency Physicians in the areas of clinical autonomy, relationships, resources, lifestyle, and challenges. | [www.ncbi.nlm.nih.gov/pubmed/8285952](https://www.ncbi.nlm.nih.gov/pubmed/8285952) |
| Physician Wellness Index | Medical professionals | Evaluate the dimensions of distress leading to burnout additionally, wellness and well-being. | [www.mededwebs.com/well-being-index](http://www.mededwebs.com/well-being-index) |
| Jefferson Scale of Patient’s Perception of Physician Empathy | Patients | Evaluates physician empathy from a patient’s perspective and can be used for assessing patient outcomes. | [www.jefferson.edu/university/skmc/research/research-medical-education/TheJeffersonScales.html](https://www.jefferson.edu/university/skmc/research/research-medical-education/TheJeffersonScales.html) |

Table 2. Depression and Suicide Risk Assessment and Prevention Resources.

| Title | Web site or resource |
| --- | --- |
| National Suicide Prevention Hotline | <https://suicidepreventionlifeline.org/>  1-800-273-8255  Text HOME to 741741 for 24 hr Crisis Text line |
| Depression Assessment Tool | <https://screening.mentalhealthamerica.net/screening-tools?show=1&ref=http%3A%2F%2Fwww.mentalhealthamerica.net%2Fconditions%2Ftake-depression-screening> |
| Tools for getting help | <http://wellmd.stanford.edu/get-help.html> |
| Centers for Disease Control (CDC) | <https://www.cdc.gov/violenceprevention/suicide/resources.html> |
| National Institutes of Mental Health | <https://www.nimh.nih.gov/health/topics/suicide-prevention/index.shtml> |
| Suicide Prevention Resource Center | <https://www.sprc.org/> |
| Suicide Prevention Resources | <https://themighty.com/suicide-prevention-resources/> |
| To Write Love on Her Arms | <https://twloha.com/find-help/local-resources/> to find local resources |
| International Association for Suicide Prevention | <https://www.iasp.info/index.php> |
| American Foundation for Suicide Prevention | <https://afsp.org/find-support/>    *<https://afsp.org/our-work/education/healthcare-professional-burnout-depression-suicide-prevention/> |
| Suicide Awareness Voices of Education | <https://save.org/> |
| Suicide Sucks | <https://suicidesucks.com/?gclid=EAIaIQobChMI7snTldeO3QIVkABpCh22mQS-EAMYASAAEgJeBfD_BwE> |
| Action Alliance | <http://actionallianceforsuicideprevention.org/resources> |
| Speaking of Suicide | <https://www.speakingofsuicide.com/resources/> |
| Accreditation Council for Graduate Medical Education (ACGME) | <https://www.acgme.org/What-We-Do/Initiatives/Physician-Well-Being/Resources> |
